# ColorTree: a batch customization tool for phylogenic trees

**DOI:** 10.1186/1756-0500-2-155

**Published:** 2009-07-31

**Authors:** Wei-Hua Chen, Martin J Lercher

**Affiliations:** 1 Bioinformatics, Heinrich-Heine University Duesseldorf, 40225, Germany; 2 European Molecular Biology Laboratory (EMBL), Meyerhofstrasse 1, 69117 Heidelberg, Germany

## Abstract

**Background:**

Genome sequencing projects and comparative genomics studies typically aim to trace the evolutionary history of large gene sets, often requiring human inspection of hundreds of phylogenetic trees. If trees are checked for compatibility with an explicit null hypothesis (e.g., the monophyly of certain groups), this daunting task is greatly facilitated by an appropriate coloring scheme.

**Findings:**

In this note, we introduce ColorTree, a simple yet powerful batch customization tool for phylogenic trees. Based on pattern matching rules, ColorTree applies a set of customizations to an input tree file, e.g., coloring labels or branches. The customized trees are saved to an output file, which can then be viewed and further edited by Dendroscope (a freely available tree viewer). ColorTree runs on any Perl installation as a stand-alone command line tool, and its application can thus be easily automated. This way, hundreds of phylogenic trees can be customized for easy visual inspection in a matter of minutes.

**Conclusion:**

ColorTree allows efficient and flexible visual customization of large tree sets through the application of a user-supplied configuration file to multiple tree files.

## Findings

### Background

Studies in comparative genomics, e.g., analyzing protein family evolution [1-3] or lateral gene transfers [4-7], typically generate large sets of phylogenic trees. Visual inspection of these trees is often necessary, as automated algorithms are not yet sufficiently flexible and reliable [8,9]. The aim of such analyses is often to check for consistency with given null hypotheses (e.g., the clustering of gene copies from known monophyletic groups). This task is often simplified by manual customization of the trees prior to inspection. Customizations usually involve changes of foreground and background colors of specific labels, line-width and color of associated branches, and other aspects of a phylogenic tree. The majority of existing tree viewing programs allow the customization of one or a few opened trees within reasonable time; few also allow to save and re-open customized results [10,11]. However, such manual customization becomes time-consuming and error prone for large trees (the tree of life or the phylogenic tree of NCBI taxonomy, for example) or large tree numbers. In some modern tree-editors published recently, TreeDyn [12] and Dendroscope [13] for example, scripting and command-line consoles are introduced to tackle the problem; in both program, users can manipulate leaves and nodes through a command-line window (the console). By using TreeDyn, user can even save their commands into script files and re-apply them to other tree files afterwards. The advantage of such implementation is that the manual customization jobs are greatly facilitated in a semi-automatic way; however, the disadvantages are also obvious: users have to learn yet some other languages (although both are as simple as plain English and easy to learn for those who had programming experiences) and it's still difficult to apply the same set of commands to multiple tree-files.

Here we introduce a new program, ColorTree, which quickly and automatically customizes phylogenic trees based on a user-supplied customization file. Results are saved in a format that can be read by Dendroscope [13], a powerful tree viewer and editor freely available from its authors . The advantages of ColorTree over existing customization methods are: (1) It is a standalone program that can be run from the command-line, making it ideally suited for batch use; (2) customized results are saved for further viewing and editing; and (3) the user-supplied configuration files, based on pattern matching logic, guarantee the stability and flexibility of customization results.

### Program overview

ColorTree takes two input text files, a tree file in any of the "Newick" and "NEXUS" formats, and a user-defined configuration file detailing the desired customizations. Input tree files may contain multiple phylogenic trees, delimited by ';'. Bootstrap scores on input tree branches are preserved.

Each line of the configuration file specifies one individual customization command. Each consists of five tab-delimited columns, specifying:

• how the keyword will be searched in branch labels

• the keyword to be searched in branch labels

• foreground color to be applied to branches and node labels

• background color to be applied node labels

• line width to be applied to branches

The first two columns are obligatory, while the other three columns can be left blank. Detailed descriptions of the configuration file, as well as instructions for its generation, are available in the software package.

**Figure 1:**
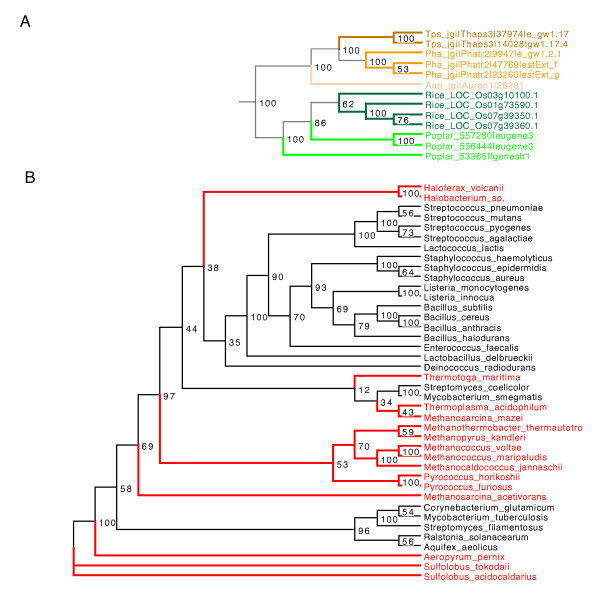
**Samples of a customized phylogenic tree**. Example plots of customized phylogenic trees. Please note that the customized output of ColorTree is displayed by Dendroscope [13].

To customize trees in the input file, terminal node labels of each tree are searched using the user-supplied keywords. Four ways of searching are supported: "prefix", "suffix", "complete", and "contain". The user-defined "background color" will be applied to all matching labels, "branch width" will be applied to the branches that directly connect to the corresponding terminal nodes, and "foreground color" to both labels and directly connecting branches. When all descendant terminal nodes of any internal node have the same color, all intervening branches will also receive that color. This is particularly useful to find the common ancestor of a group of genes, or to pinpoint the separation of two clades during evolution (see examples in Figure 1).

Customized tree(s) are saved in ".dendro" format, which can be viewed and further edited by Dendroscope [13]. It should be pointed out that Dendroscope provides a range of tree customization methods, but these have to be applied to individually opened tree files and tends to be time-consuming.

### Examples of customized trees

In a genome sequencing project, evolutionary paths of selected protein families in multiple organisms were investigated. This required visual inspection of several hundred gene families, each containing orthologous genes from different organisms as well as paralogous copies within organisms. Using ColorTree, hundreds of phylogenic trees can be customized within a few hours on a standard desktop computer. All customized trees were then visually inspected in Dendroscope.

Several examples of customized trees are shown in Figure 1. Figure 1a shows a phylogenetic tree for the sorbitol transporter protein. This represents a typical scenario of lineage-specific gene duplications. An ancestral sorbitol transporter gene is found in the common ancestor of green plants (highlighted in green) and brown algae (highlighted in brown). After the separation of green and brown algae, the ancestral gene remained single copy in brown algae, duplicating only in the terminal branches. While there are also duplications specific to rice and poplar, one duplication is evident before the separation of these two species.

Figure 1b shows a phylogenic tree for the glutamine synthetase protein, which was adopted from [5]. Species of archaea (red) and bacteria (black) are intermingled. The tree is thus incompatible with the accepted monophyly of the two kingdoms. If the tree faithfully reflects the evolutionary history of the gene, this would indicate possible lateral gene transfers (LGT) between bacteria and archea. Thus, visual inspection using ColorTree and Dentroscope is a simple and intuitive way to identify certain types of inconsistencies in genetic data. However, users may also wish to look at alternative, sophisticated methods to detect such inconsistencies, e.g., Neighbour-Net [14] or the 'tree-of-tree' approach [15].

## Availability and requirements

The program described in this note is freely downloadable from . ColorTree is written in PERL and should run on any platform running PERL and BioPerl. To facilitate users who don't have programming experience or have no PERL pre-installed, we also provide pre-packed executables that can run on computers without PERL and BioPerl modules.

Requirements: 5.8 or latter version of PERL program  and 1.4 or latter version of BioPerl module .

## Competing interests

The authors declare that they have no competing interests.

## Authors' contributions

WHC conceived the project and implemented TreeColor in consultation with MJL. WHC and MJL wrote the manuscript.
